# Type IV collagen reduces MUC5AC secretion in the lungs of ovalbumin-sensitized mice

**DOI:** 10.3389/fphar.2022.851374

**Published:** 2022-09-16

**Authors:** Jun Iwashita, Hikari Maeda, Momo Ishimura, Jun Murata

**Affiliations:** Faculty of Bioresource Sciences, Akita Prefectural University, Akita, Japan

**Keywords:** MUC5AC, MUC5B, mucin, asthma, collagen

## Abstract

Mucin 5AC (MUC5AC) is excessively secreted in the respiratory tract of patients with asthma. Suppressing this secretion is important for improving the air passages, which facilitates easy breathing. We have previously reported that the addition of type IV collagen, a typical extracellular matrix (ECM) protein, to the culture medium for human cell lines and primary cells reduced MUC5AC secretion. In this report, we further investigated the effect of type IV collagen on MUC5AC secretion *in vivo*. We employed ovalbumin (OVA)-sensitized mice to model of asthma and exposed them to type IV collagen to verify the reducing effect of MUC5AC *in vivo*. The amount of MUC5AC in bronchoalveolar lavage fluid was examined after nebulization of type IV collagen. Hypersecretion of MUC5AC of the OVA-sensitized mice was suppressed by type IV collagen exposure in a time- and dose-dependent manner. Furthermore, type IV collagen exposure to OVA-sensitized mice decreased integrin α2 and β1 expression in the lungs and increased the levels of Akt and extracellular signal-regulated kinase (ERK) phosphorylation in the trachea. These results suggest that type IV collagen suppresses MUC5AC hypersecretion *via* modulating integrin expression and Akt/ERK phosphorylation in the respiratory tract of the OVA-sensitized mice.

## Introduction

The surface of the human respiratory tract is covered with a mucus layer that provides essential biological defense against foreign irritants (Aikawa et al., 1992; Vestbo, 2002). The mucus layer contains mucin protein as its main component. To date, at least 20 mucin subtypes have been identified in human organs and are expressed in specific organs depending on the subtype (Voynow et al., 2006). For example, both mucin 5B (MUC5B) and mucin 5AC (MUC5AC) are mainly secreted in the respiratory tract. MUC5B is produced in the normal respiratory tract and is important for biological defense (Roy et al., 2014). In contrast, MUC5AC is overproduced 40–200 times in the lungs of patients with bronchial asthma (Aikawa et al., 1992; Fahy, 2002; Vestbo, 2002; Rogers, 2004; Rose and Voynow, 2006; Wang et al., 2008). Bronchial asthma is a common clinical respiratory syndrome caused by viruses and environmental allergens and is characterized by bronchial hyper-responsiveness and inflammation (Holgate, 2008). Bronchial asthma induces reversible airflow limitation, mucus hypersecretion, and structural remodeling of the lung and trachea; however, its pathogenesis remains unclear (Hehua et al., 2017; Qu and Zhang, 2017).

MUC5AC secretion is regulated by proinflammatory cytokines, cell adhesion molecules such as cadherins, and kinases. These regulate MUC5AC secretion and induce hypersecretion of mucus in the lung epithelial cells (Chen et al., 2003; Rogers, 2003; Song et al., 2003; Iwashita et al., 2011; Iwashita et al., 2014). Signaling from various stimulations activates the epidermal growth factor receptor (EGFR), extracellular signal-regulated kinase (ERK) pathway, and Akt kinase intervening pathway. Consequently, the transcription factor, nuclear factor-κB is activated, in turn increasing MUC5AC secretion (Iwashita et al., 2014; Huang et al., 2017; Ito et al., 2019). Previous studies have suggested that the Akt pathway activation in the human lung epithelial cell line NCI-H292 suppresses MUC5AC secretion, whereas the activation of the MEK/ERK pathway promotes MUC5AC secretion (Iwashita et al., 2014).

The epithelial cells in the lungs and trachea are supported by the extracellular matrix (ECM), which contains several proteins such as laminins, fibronectins, and collagens (Ingber et al., 1994; Coppock et al., 2007; Wilhelmsen et al., 2007; Yang et al., 2007; Segal et al., 2008; Osei et al., 2020). Type IV collagen (Col4) plays a key role in cell support and cell–cell communication. We have previously reported that some ECM proteins regulated MUC5AC expression. For example, MUC5AC secretion decreased in both NCI-H292 human lung cancer cells and human primary asthmatic lung cells, which cultured with Col4. In contrast, MUC5AC secretion increased in cells cultured with laminin (Iwashita et al., 2010; Ito et al., 2019). However, the role of the signaling pathways from ECM proteins in regulating MUC5AC and MUC5B secretion has not yet been clarified *in vivo*.

Integrins are receptors for ECM proteins; they are heterodimeric membrane-bound receptors composed of noncovalently bound α and β subunits (Hynes, 2002; Barczyk et al., 2010). Currently, at least 18 α subunits and 8 β subunits have been identified (Barczyk et al., 2010). Integrin α2β1 is a combination that recognizes Col4 in NCI-H292 cells (Setty et al., 1998). Formerly, we have reported that cell attachments and the expression level of integrin β1 subunit might regulate MUC5AC secretion (Iwashita et al., 2013). Furthermore, a previous study revealed that the overexpression of integrin β1 reduced MUC5AC expression but not MUC5B expression in NCI-H292 cells (Iwashita and Murata, 2021). However, the role of integrin β1 subunits in regulating MUC5AC secretion *in vivo* remains undefined.

In this study, the effect of Col4 exposure on MUC5AC secretion in the lungs was investigated in ovalbumin (OVA)-sensitized mice. Further, we analyzed several integrins that connect the lung epithelial cells to ECM proteins and the Akt and ERK activities that regulate MUC5AC secretion in the airways of OVA-sensitized mice.

## Materials and methods

### Animal model and groups

Six-week-old female Hos:HR-1 mice weighing 18–20 g were purchased from Hoshino Laboratory Animals, Inc. (Ibaraki, Japan). In this mouse model, the Hrhr/Hrhr gene was introduced into intron 6 of the Hr gene (chromosome 14). The Hr gene encodes a protein whose function has been linked to hair growth. The Hos:HR-1 mice exhibit a nude mouse-like morphology. However, the immune system is normal, and the overexpression of MUC5AC induced by OVA sensitization is more pronounced in Hos:HR-1 mice than in C57BL/6 mice. The animals were maintained under OVA-free conditions. The protocol was approved by the Animal Ethics Committee of the Akita Prefectural University (Permit Number: 20-11). All surgeries were performed under anesthesia induced by isoflurane (Wako, Osaka, Japan), and all possible efforts were made to minimize the suffering of the mice. This study comprised the following 10 groups (each group, *n* = 5): naive control group, groups exposed to 10, 5, 0.5, or 0 mg of Col4 exposed OVA administrated group for 4 and 24 h.

### Preparation of OVA-sensitized mice

**FIGURE 1:**
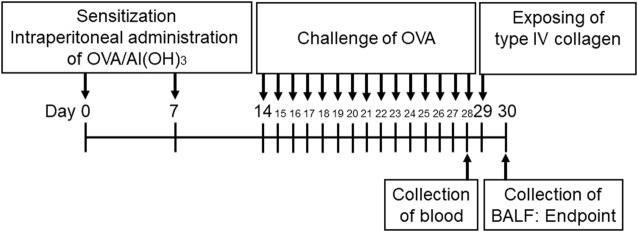
Timetable for establishing ovalbumin (OVA)-sensitized mice. The mice were sensitized *via* intraperitoneal injections of OVA on days 0 and 7. The animals then received aerosolized OVA challenge on days 14–28 using a nebulizer. The mice were exposed to Col4 on day 29 and were euthanized on day 30 after 24 h of Col4 exposure. The control mice and the mice treated with 0 mg of Col4 were exposed to PBS alone at the same time.

The mice were sensitized intraperitoneally using 0.5 mg OVA (Grade V, Wako) with 0.05 g aluminum hydroxide dissolved in 5 ml of phosphate buffered saline (PBS) by shaking the solution for 30 min at room temperature. Subsequently, 200 µl of this solution was injected into each mouse. Five mice were included in each group. A dose of 20 µg of OVA was administered on days 0 and 7. The control mice were injected and challenged with PBS alone. The mice were challenged *via* aerosol nebulization using a nebulizer (Koshin, Saitama, Japan) with 1% OVA (wt/vol) for 30 min each day from day 14 to day 28 in a 12.4 L chamber. The control mice were received PBS in both steps. The mice were euthanized using an overdose of isoflurane (Wako) on day 30. The entire procedure for the preparation of mice was summarized and illustrated in Figure 1 (Wagers et al., 2004; Zosky and Sly, 2007; Yu and Chen, 2018).

### Quantifying the anti-OVA antibodies in blood samples using the enzyme-linked immunoassay method

Blood samples were collected from the tail vein of mice on day 28, and anti-OVA antibodies were detected using Anti-Ovalbumin IgG1 (mouse) ELISA Kit (Cayman Chemical, MI, United States). The antibody levels were measured according to the manufacturer’s instructions using Infinite F200 Pro microplate reader (Tecan, Grödig, Austria).

### Col4 exposure

Five milliliters of PBS was added to 10, 5, 0.5 or 0 mg of Col4 taken from human placenta (C5533, Sigma Aldrich, St. Louis, MO, United States) to dissolve it. The PBS containing Col4 was then immediately exposed to five mice using a nebulizer in a 12.4 L chamber for 30 min on day 29.

### Collection of the bronchoalveolar lavage fluid sample from mice

This experiment was conducted according to a previously reported thesis (Van Hoecke et al., 2017). To summarize, the skin of the mouse was cut open using scissors to expose the muscle around the trachea on day 30. The middle part of the exposed trachea was then punctured with a 26-gage needle (Terumo, Tokyo, Japan), followed by the insertion of a 27-gauge catheter (NIPRO, Tokyo, Japan) approximately 0.5 cm into the trachea. The catheter was fixed by tying the trachea around it using a cotton thread. A 1 ml tuberculin syringe was loaded with 500 µl of sterile PBS containing 100 µM EDTA and was connected to the catheter. The PBS was injected into the lung and aspirated. This injection and aspiration step was repeated 3 times, and the recovered lavage fluid samples were stored as bronchoalveolar lavage fluid (BALF) samples at 4°C.

### MUC5AC and MUC5B protein measurement using the dot-blot method

The collected BALF samples were diluted at a ratio of 1:500 with Tris-buffered saline (20 mM Tris, pH 7.4, 150 mM NaCl) with 0.1% sodium dodecyl sulfate (SDS), and 10 µl of the solution was blotted onto an Immobilon membrane (Millipore, Temecula, CA, United States) using a Dot Blot Hybridization Manifold (48 well, SCIE PLAS, Cambridge, United Kingdom). When measuring cellular MUC5AC and MUC5B levels in the lungs, the whole lungs after BALF collection were added to 250 µl of 4 M guanidine hydrochloride (Nacalai Tesque, Kyoto, Japan) with 1 mM phenylmethylsulphonylfluoride (Wako). Samples were prepared in quantity. The lungs were homogenized, and 5 µl of samples were applied onto an Immobilon membrane. The membrane was treated with a western blot blocking buffer (T7131A, Takara, Tokyo, Japan) in Tris-buffered saline (20 mM Tris, pH 7.4, 150 mM NaCl) with 0.1% Tween 20 (TBS-T) for 12 h at 4°C. The membrane was then incubated with mouse anti-MUC5AC antibody (MS145-P1, 1:2000 in western blot blocking buffer, Neomarkers, Fremont, CA, United States) or mouse anti-MUC5B antibody (ab77995, 1:2,000 in 4% skim milk, Abcam, Tokyo, Japan) for 1 h. The membrane was washed 5 times with TBS-T for 5 min each and then incubated with rabbit anti-mouse IgG (H + L) (1:2,000 in 4% skim milk, NA931V, GE Healthcare, Buckinghamshire, United Kingdom) for 1 h. After washing the membrane five times, the enzymatic reactions were detected using Luminata Forte western horseradish peroxidase (HRP) substrate (WBLUF0500, Millipore) and a ChemiDoc image analyzer (Bio-Rad).

### Immunoblot detection of integrin α2, β1, β3, glyceraldehyde-3-phosphate dehydrogenase, phosphorylated ERK, total ERK, phosphorylated Akt total Akt, β actin, MUC5AC, and MUC5B

When measuring cellular protein levels in the lung and trachea tissues, the tissue samples after BALF collection were added to 500 μl of 1 X Laemmli sample buffer (161-074, Bio-Rad, Tokyo, Japan). The cellular proteins were electrophoresed on a 7.5% or 10% SDS PAGE gel using an IEP-1010 electrophoresis apparatus (AXEL, Tokyo, Japan). The samples were then blotted onto a nitrocellulose membrane (Hybond ECL, GE Healthcare) using a BE351 transfer apparatus (Bio Craft, Tokyo, Japan). The membrane was treated with western blot blocking buffer in TBS-T for 12 h at 4°C and incubated with rabbit anti-integrin β1 polyclonal antibody (4706S; Cell Signaling Technology Japan, Tokyo, Japan), mouse anti-integrin α2 (C9) (sc74466; Cosmo Bio, Tokyo, Japan), rabbit anti-integrin β3 (4702; Cell Signaling Technology Japan, Tokyo, Japan), rabbit anti-phospho EGF (active) receptor (2220S; Cell Signaling Technology Japan, Tokyo, Japan), rabbit anti-phosphorylated (active) ERK1/2 antibody (GTX24819, Funakoshi, Tokyo, Japan), or rabbit anti-ERK 1/2 antibody (V1141, Promega, Madison, WI, United States), rabbit anti-phopho (active) Akt (S473) (4058L; Cell Signaling Technology Japan), or rabbit anti-Akt (GTX121937; Funakoshi, Tokyo, Japan), at a dilution of 1:2,000 in western blot blocking buffer for 1 h. The membrane was washed 5 times with TBS-T for 5 min each and then incubated with anti-rabbit IgG antibody conjugated with horseradish peroxidase (W4011, Promega) at a dilution of 1:2,000 for 1 h. After washing the membrane five times, the enzymatic reaction was detected using Luminata Forte Western HRP Substrate and a ChemiDoc image analyzer. Cellular β actin and GAPDH were detected as a loading controls using rabbit anti-β actin antibody (A5316, Sigma) and anti-GAPDH antibody (ab9485; Abcam, Tokyo, Japan) at a 1:2,000 dilution and using anti-rabbit IgG antibody conjugated with horseradish peroxidase at the same dilution in western blot blocking buffer. Subsequently, the blotted membrane was incubated with the Restore Western Blot Stripping Buffer (21059, Thermo Fisher Scientific) for 15 min at room temperature under shaking conditions. The membrane was washed five times with TBS-T for 5 min each and then treated with western blot blocking buffer for 12 h at 4°C for reblocking. To detect MUC5AC and MUC5B protein levels in BALF samples using the immunoblot method, a 10% volume of 10X loading buffer [0.5X TAE, glycerol (50%), bromophenol blue (0.25%), sodium dodecyl sulfate (SDS) (1%)] was added to each collected BALF sample which was denatured in 6 M urea (Ramsey et al., 2016). Next, 10 μl of the solution was run on a 0.8% agarose gel with 1× TAE (40 mM Tris-acetate, 1 mM EDTA)-0.1% SDS buffer. This process was performed using an Mupid2 electrophoresis apparatus (Cosmo-Bio, Tokyo, Japan), and the proteins were vacuum blotted onto an Immobilon membrane (Millipore) using a Dot Blot Hybridization Manifold (DHM-96, SCIE PLAS). Finally, MUC5AC and MUC5B in BALF were detected in a similar manner as that of the dot-blot method, but the detection of MUC5B was performed with rabbit anti-MUC5B antibody (GTX17503, 1:2,000 in 4% skim milk, Genetex, Irvine, CA, United States) and with anti-rabbit IgG antibody conjugated with horseradish peroxidase (W4011, Promega).

### Lung sectioning

The lungs were removed from the mice with 4% formaldehyde (Mildform 10N, Wako) for tissue fixation for 1 week at 4°C. Each sample was provided to the Akita University for preparing sections, and Alcian blue and periodic acid–Schiff stains were used for the histological analysis. Briefly, the fixed samples were preliminary degreased in water for 10 min and 70% ethanol was added and stored for 2 h at room temperature. The samples were treated 6 times with 100% ethanol for 1.5 h, four times with chloroform for 1.5 h, and 4 times with paraffin for 1.5 h. The fixed tissue samples were cut into sections with an approximate thickness of 5 μm and mounted on slides using a microtome (REM-710, Yamato Kohki, Saitama, Japan). Representative sections were stained with 1% Alcian blue (pH 2.5, 8GX, Sigma) solution for 30 min and potassium hydroxide (0.5%) for 10 min. The sections were treated with Cold Schiff’s Reagent for 10 min and Mayer’s hematoxylin solution for 30 min. The slides were observed under a microscope (BZ-9000, Keyence, Tokyo, Japan).

### Morphology analysis using imaging software

The obtained digital images were analyzed using an image analysis tool, NIH ImageJ software (Schneider et al., 2012). The software is open-source and publicly available for download (https://fiji.sc/). For quantitative analysis, we determined the cell areas using the Rectangle selection tool and selected a region with no staining to identify the background value. The data of five pictures, which were obtained at the same time interval, were averaged for each sample.

### Statistical analysis

The differences between the experimental groups were statistically analyzed using analysis of variance (ANOVA) and two-tailed unpaired student’s t-test. ANOVA was used for comparisons among more than two groups. ANOVA and student’s t-test were performed and the *p-*values were calculated using Microsoft Office professional plus 2016 Excel. A *p-value of <0.05 was considered statistically significant. All experiments using BALF samples were performed at least 3 times, and the representative results were presented.

## Results

### Anti-OVA antibodies were increased in blood samples collected from OVA-sensitized mice

**FIGURE 2:**
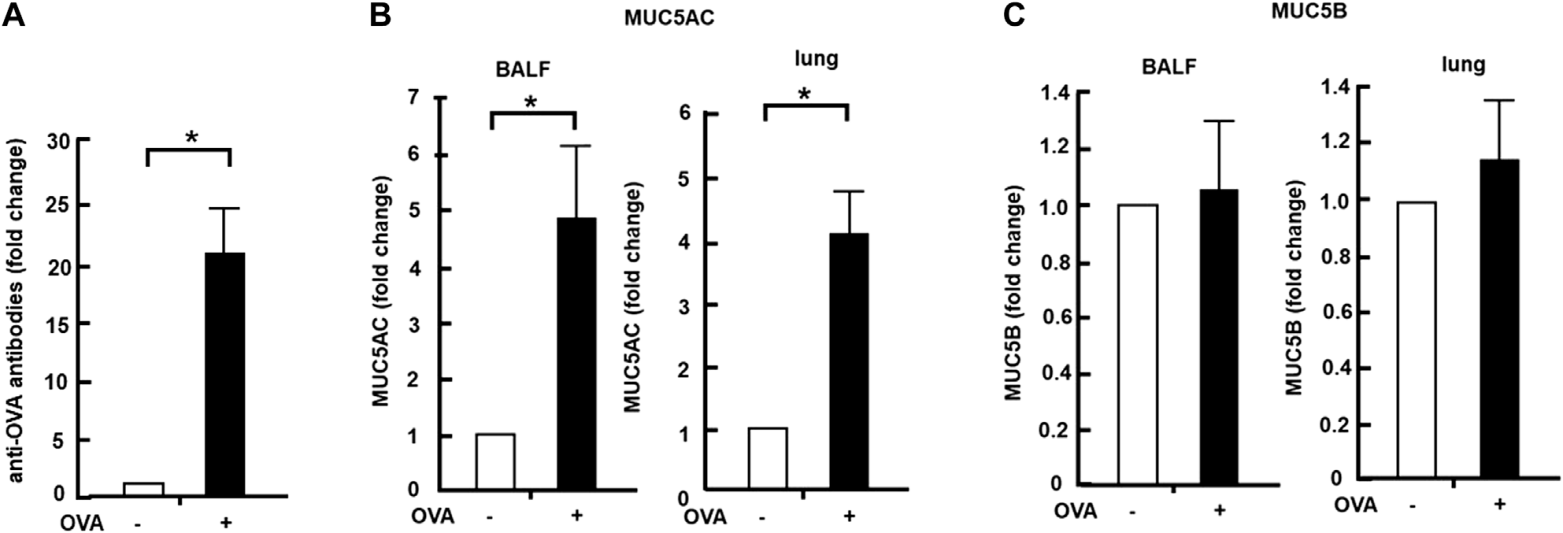
Effects of Col4 on cellular and secreted mucin 5AC (MUC5AC) and mucin 5B (MUC5B) levels in ovalbumin (OVA)-sensitized mice. **(A)** Blood samples were collected from the OVA-sensitized (+) and control mice groups (−), and the amount of antibodies to OVA present in the blood sample was detected using the enzyme-linked immunoassay method. **(B)** MUC5AC levels in bronchoalveolar lavage fluid (BALF) or lung tissue samples were detected using the dot-blot method in the OVA-sensitized mice (+) and unsensitized control mice (−). **(C)** MUC5B levels in BALF or lung tissue samples were detected using the dot-blot method in the OVA-sensitized mice (+) and unsensitized control mice (−). The MUC5AC and MUC5B protein levels in the lung tissue samples were normalized to the GAPDH levels before analysis. Fold changes were based on the ratio between the values [mean ± standard deviation (SD), *n* = 5]. A student’s t-test was used to obtain the *p*-values. Asterisks indicate statistical significance, *p < 0.05. Representative results of three independent experiments are presented in this figure.

OVA-sensitized mice were established using the OVA-sensitizing method (Figure 1). Blood samples were collected from both OVA-sensitized and control groups. The level of antibodies against OVA present in the blood sample was detected using the ELISA method. The blood samples of the OVA-sensitized mice group showed a 21-fold increase in the anti-OVA antibody level compared with those of the control group (Figure 2A). The BALF and lung tissue samples were then collected from the OVA-sensitized mice, and the levels of MUC5AC and MUC5B were quantified using the dot-blot method. The OVA-sensitized mice group showed a 4-fold increase in MUC5AC secretion (BALF) and cellular protein levels in the lungs compared with the control group (Figure 2B). However, MUC5B secretion and cellular protein levels in the lungs remained unchanged in the OVA-sensitized mice (Figure 2C).

### Col4 decreased the amount of MUC5AC in BALF and in the lungs in a dose- and time-dependent manner

**FIGURE 3:**
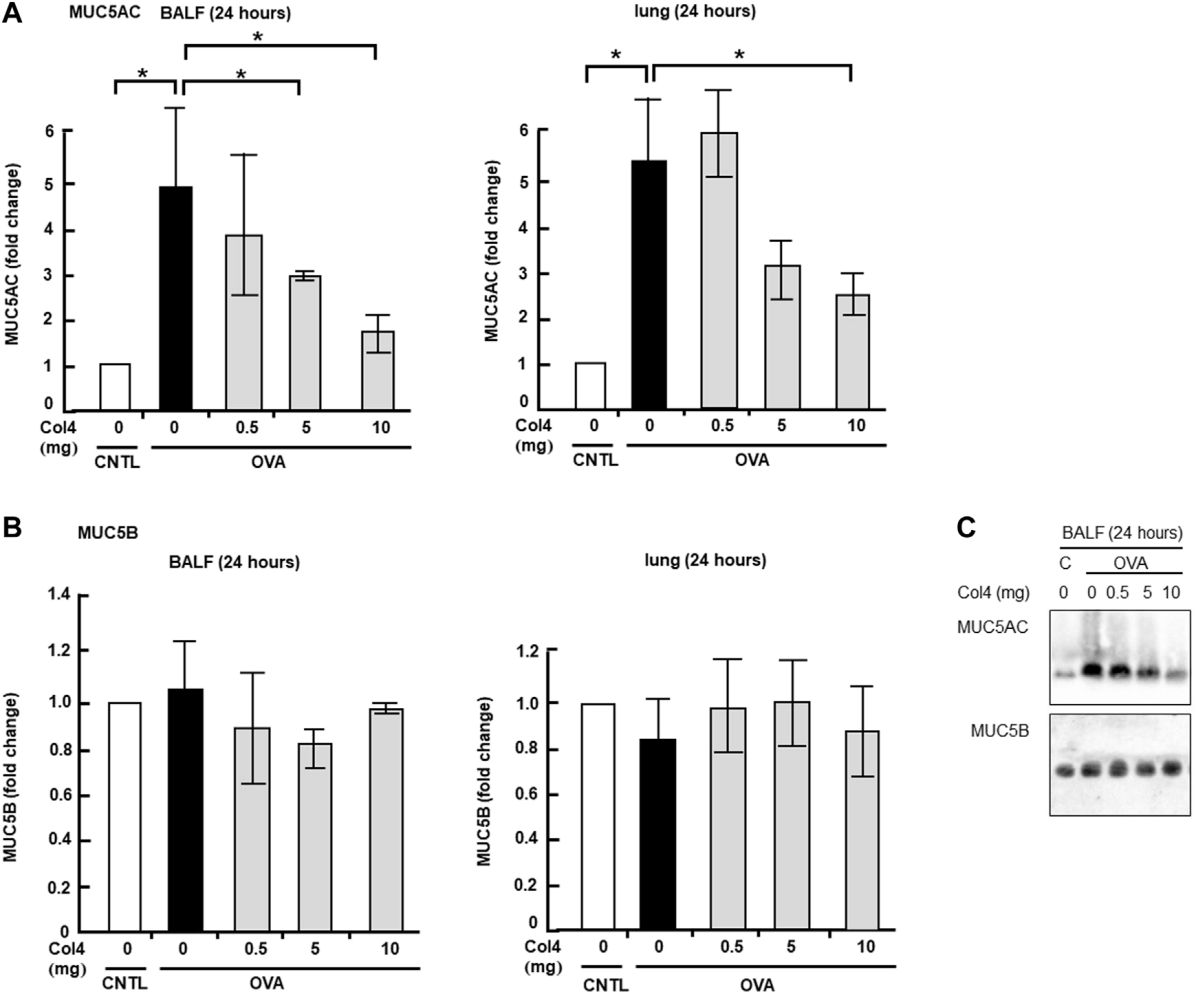
Effects of Col4 on cellular and secreted mucin 5AC (MUC5AC) and mucin 5B (MUC5B) levels in ovalbumin (OVA)-sensitized mice. **(A)** The control mice (CNTL) and OVA-sensitized mice (OVA) were exposed to Col4 at various concentrations (0–10 mg). After 24 h of Col4 exposure, MUC5AC levels in bronchoalveolar lavage fluid (BALF) or lung tissue samples were detected using the dot-blot method and measured. **(B)** The OVA-sensitized mice were exposed to Col4 at various concentrations (0–10 mg). After 24 h of Col4 exposure, MUC5B levels in BALF or lung tissue samples were detected using the dot-blot method and measured. **(C)** The control mice (C) and OVA-sensitized mice (OVA) were exposed to Col4 at various concentrations (0–10 mg). After 24 h of Col4 exposure, MUC5AC, and MUC5B levels in BALF samples were detected using the immune-blot method with agarose gel. The MUC5AC and MUC5B protein levels in lung tissue samples were normalized to the GAPDH levels before analysis. The control mice and the mice treated with 0 mg of Col4 were exposed to PBS alone at the same time. Fold changes were based on the ratio between the values [mean ± standard deviation (SD), *n* = 5]. A one-way analysis of variance was used to obtain the *p*-values. Asterisks indicate statistical significance, *p < 0.05. Representative results of three independent experiments are presented.

The OVA-sensitized mice were exposed to aerosolized Col4, and after 24 h, BALF was collected and analyzed. The mean weights of the control and OVA-sensitized mice were 24.4 and 24.1 g, respectively. Although the amount of Col4 inhaled by each mouse was unclear, it was applied at 83, 41, or 4.1 μg per gram of the body weight for each mouse. The increase in the levels of MUC5AC in BALF samples of the OVA-sensitized mice did not show significant changes under exposure to 0.5 mg of Col4 but decreased in response to 5 and 10 mg of Col4 in the OVA-sensitized mice (Figure 3A). This decrease in MUC5AC due to exposure to 10 mg of Col4 was also observed in the lung tissue samples.

In contrast, the MUC5B cellular protein level in the lungs and its secretion did not show significant changes in the Col4-exposed OVA-sensitized mice (Figure 3B). Similar results were obtained using immunoblot analysis (Figure 3C).

**FIGURE 4:**
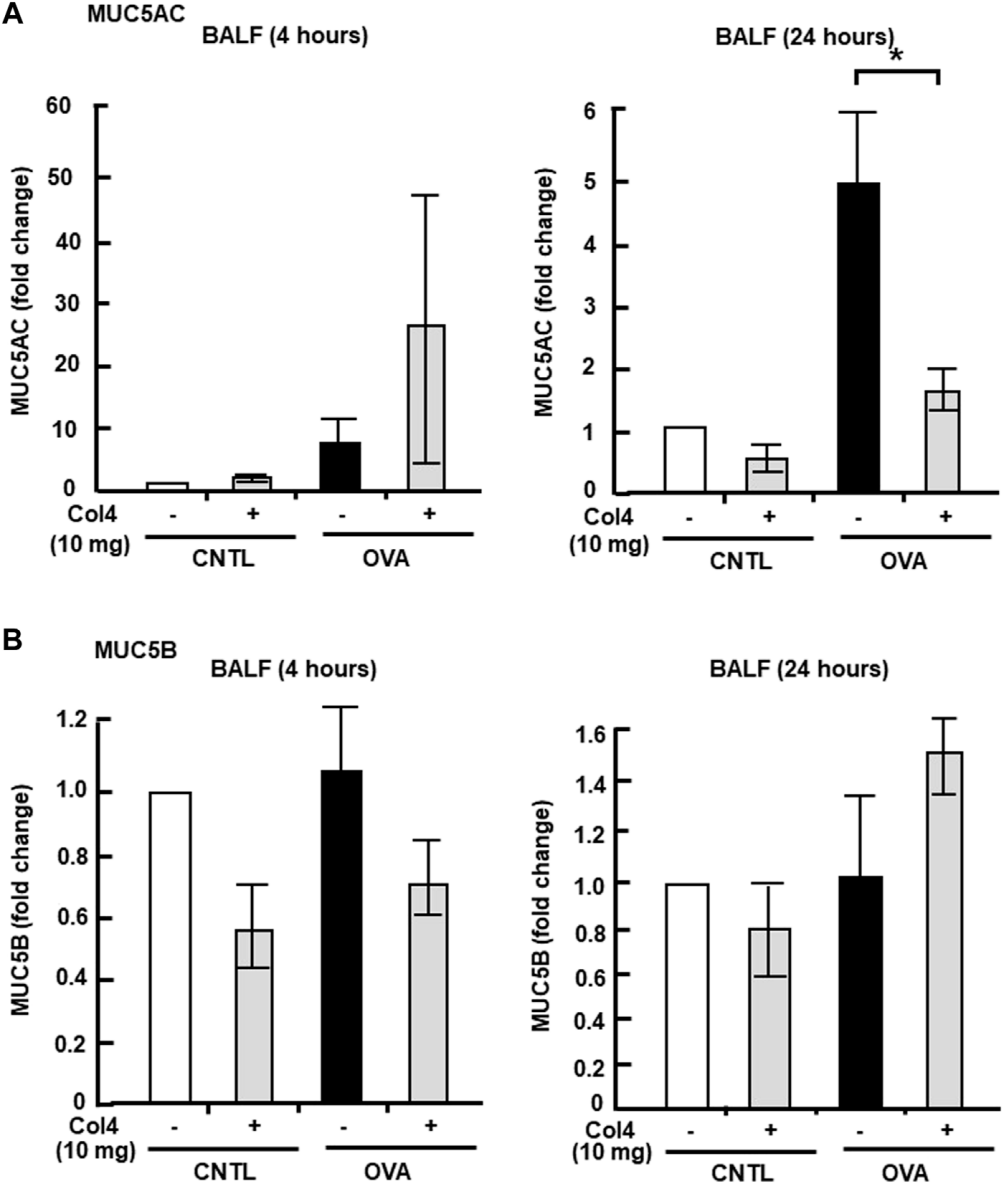
Effects of Col4 on secreted mucin 5AC (MUC5AC) and mucin 5B (MUC5B) levels in ovalbumin (OVA)-sensitized mice. **(A)** The control mice (CNTL) and OVA-sensitized mice (OVA) were exposed to Col4 (+) or PBS alone (−). After 4 and 24 h of Col4 exposure, MUC5AC levels in bronchoalveolar lavage fluid (BALF) samples were detected using the dot-blot method and measured. **(B)** The control mice and OVA-sensitized mice were exposed to Col4 (+) or PBS alone (−). After 4 and 24 h of Col4 exposure, MUC5B levels in BALF samples were detected using the dot-blot method and measured. Fold changes were based on the ratio between the values [mean ± standard deviation (SD), *n* = 5]. A one-way analysis of variance was used to obtain the *p*-values. Asterisks indicate statistical significance, *p < 0.05. Representative results for three independent experiments are presented.

**FIGURE 5:**
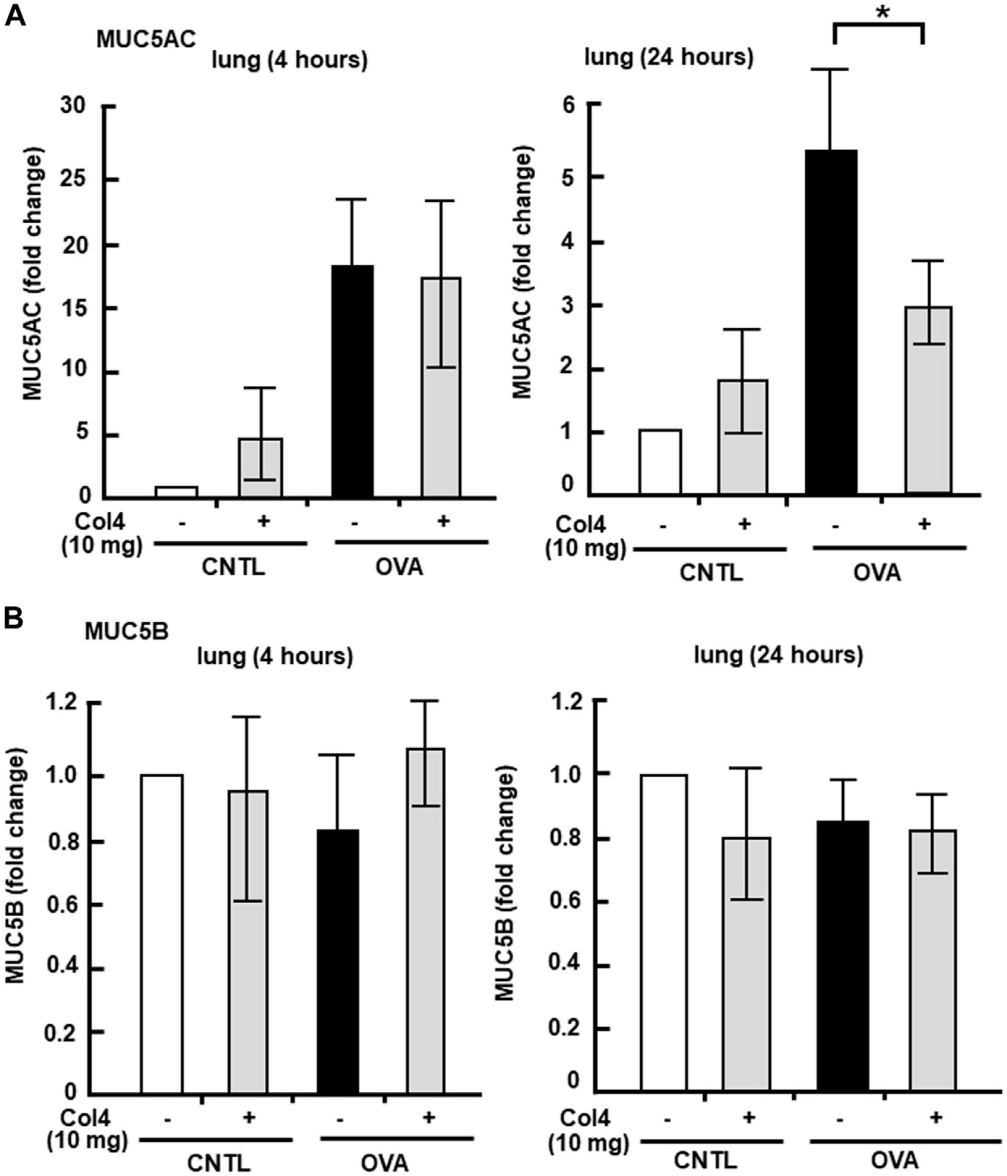
Effects of Col4 on cellular mucin 5AC (MUC5AC) and mucin 5B (MUC5B) levels in ovalbumin (OVA)-sensitized mic. **(A)** The OVA-sensitized mice were exposed to Col4 (10 mg). After 4 and 24 h of Col4 exposure, MUC5AC levels in bronchoalveolar lavage fluid (BALF) or lung tissue samples were detected using the dot-blot method and measured. **(B)** The OVA-sensitized mice were exposed to Col4 at various concentrations (0–10 mg). After 4 and 24 h of Col4 exposure, MUC5B levels in BALF or lung tissue samples were detected using the dot-blot method and measured. The MUC5AC and MUC5B protein levels in lung tissue samples were normalized to the GAPDH levels before analysis. The control mice and the mice treated with 0 mg of Col4 were exposed to PBS alone at the same time. Fold changes were based on the ratio between the values [mean ± standard deviation (SD), *n* = 5]. A one-way analysis of variance was used to obtain the *p*-values. Asterisks indicate statistical significance, *p < 0.05. Representative results of three independent experiments are presented.

MUC5AC and MUC5B expressed in BALF and in the lung tissue were analyzed at 4 and at 24 h. Reduction in MUC5AC as a result of Col4 exposure was not observed in the OVA-sensitized BALF and lungs that were collected at 4 h after Col4 exposure (Figures 4A, 5A). The amount of MUC5AC was increased in BALF and in the lungs of OVA-sensitized mice when compared with the control mice, which reduced following 10 mg of Col4 exposure for 24 h (Figures 4A, 5A). In contrast, MUC5B levels in BALF and lungs did not change significantly in Col4-exposed OVA-sensitized mice after 4 and 24 h of Col4 exposure (Figures 4B, 5B).

### Mucus in the lungs decreased following Col4 exposure

**FIGURE 6:**
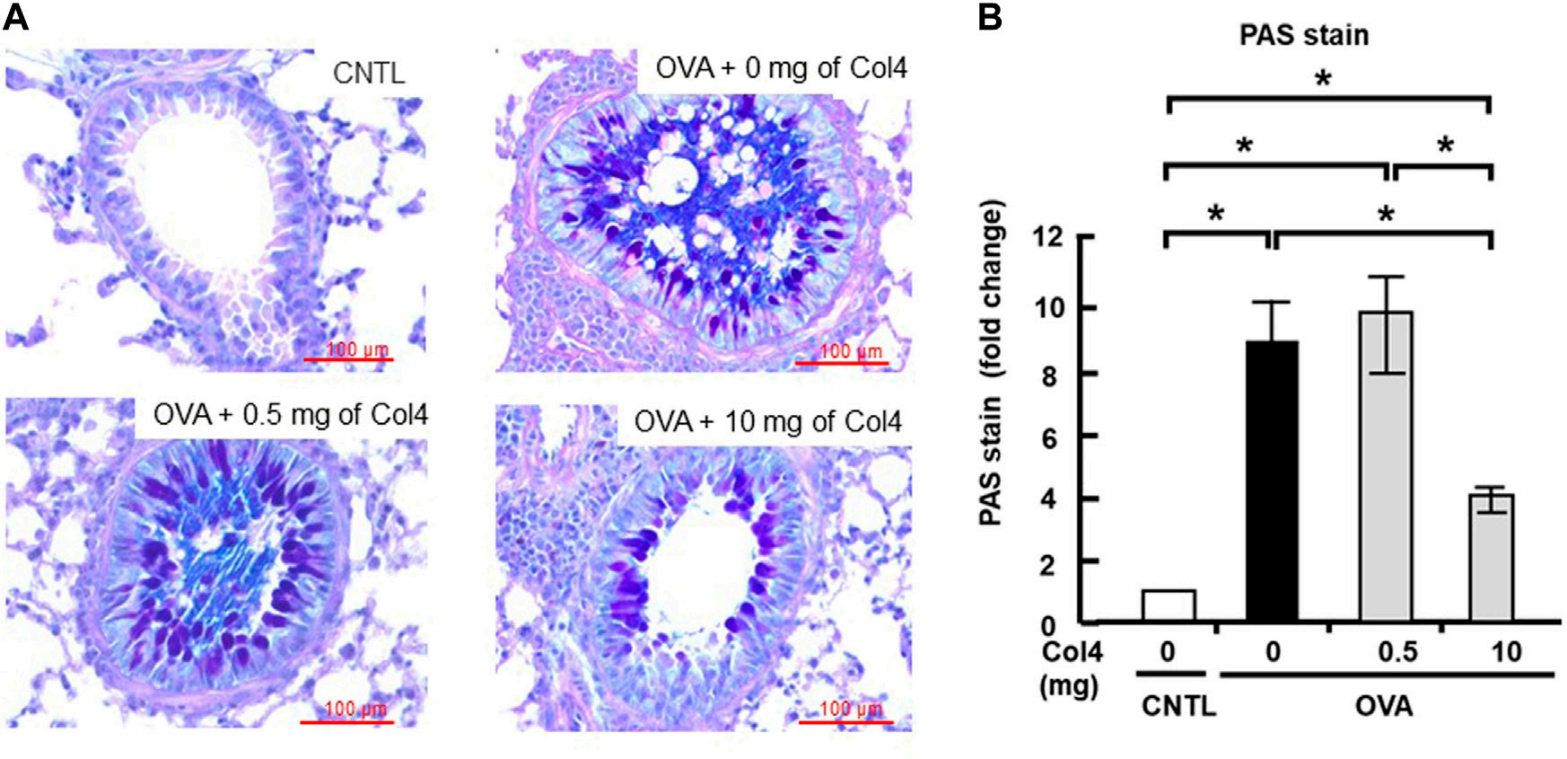
Effects of Col4 on cellular mucus levels in ovalbumin (OVA)-sensitized mice. **(A)** The OVA-sensitized mice were exposed to Col4 (0–10 mg). After 24 h of Col4 exposure, mucus in the lung tissue sections was stained with Alcian blue and periodic acid–Schiff (PAS) and observed under a microscope. The mucus was dyed in purple color. Representative results are presented. Red bars indicate 100 μm. **(B)** The staining intensity was quantified and compared using ImageJ (Fiji) with five independent images. A one-way analysis of variance was used to obtain the *p*-values. Asterisks indicate statistical significance, *p < 0.05. The control mice and the mice treated with 0 mg of Col4 were exposed to PBS alone at the same time.

The mucus in the lung tissue section was stained with Alcian blue and periodic acid–Schiff (PAS). In the lung tissue, bronchiole mucus was strongly stained by PAS in the OVA-sensitized mice, whereas stained mucus significantly decreased in 10 mg of Col4-exposed mice (Figure 6A). This change in the staining of mucus because of OVA challenge and Col4 exposure were statistically significant (Figure 6B).

### Effect of Col4 on the expression of integrin α2, β1, and β3 in the lungs and trachea

**FIGURE 7:**
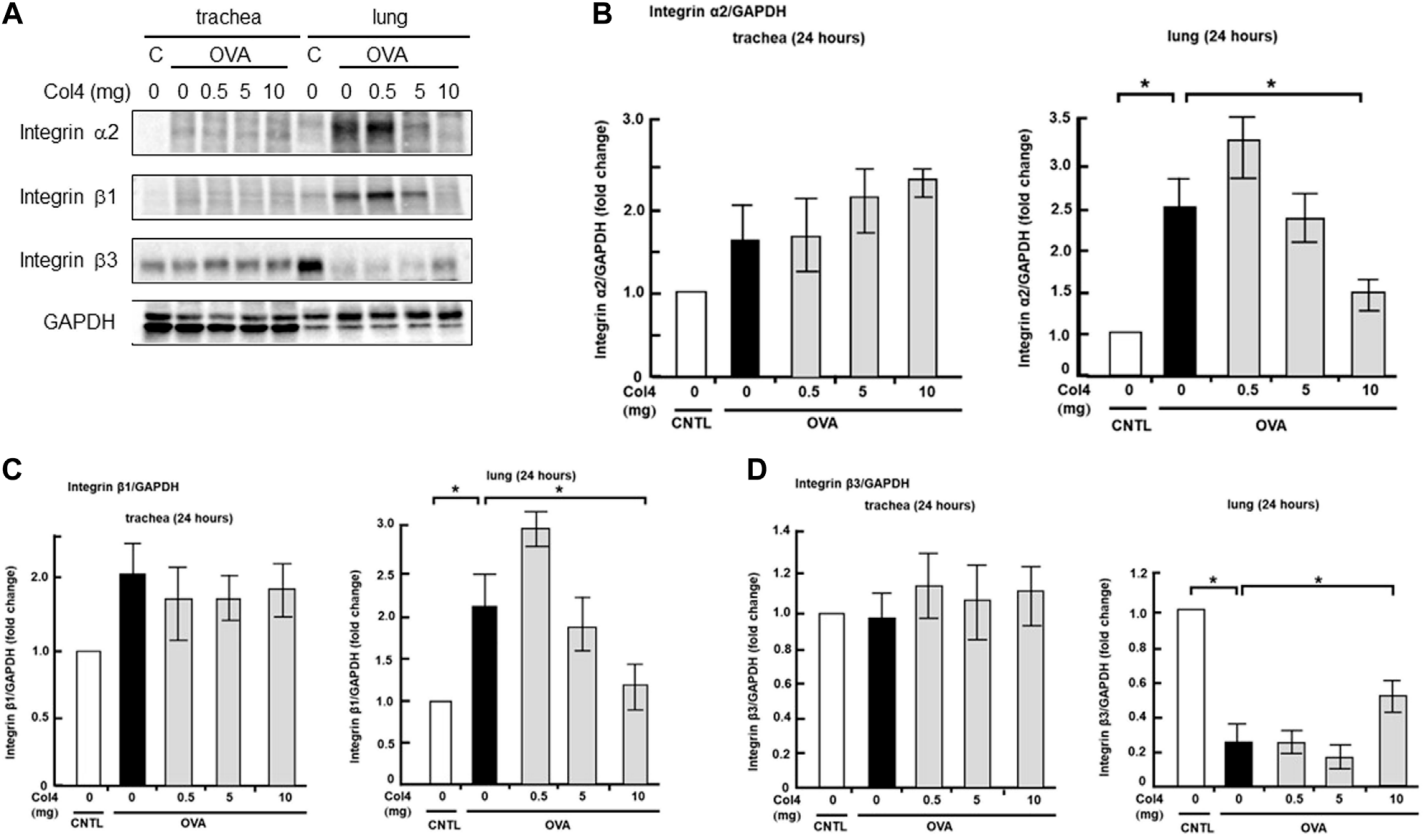
Effects of Col4 on integrin α2, β1, β3, and GAPDH protein levels in ovalbumin (OVA)-sensitized and unsensitized control mice. **(A)** The control mice (C) and OVA-sensitized mice (OVA) were exposed to Col4 at various concentrations (0–10 mg). After 24 h of Col4 exposure, the trachea and lungs were sampled and integrin α2, β1, β3, and GAPDH protein levels were detected. Graphs show the relative levels of integrin α2 **(B)**, β1 **(C)** and β3 **(D)** per GAPDH compared with the control. Representative results of three independent experiments are presented. The control mice and the mice treated with 0 mg of Col4 were exposed to PBS alone at the same time.

Integrin receptors contributed to MUC5AC expression (Iwashita and Murata, 2021). Low-level expression of integrin α2 and β1 was noted in the trachea and the expression levels did not show any changes after Col4 exposure. However, in the lung tissue, integrin α2 and β1 expressions increased in the OVA-sensitized mice and were decreased by Col4 in a dose-dependent manner (Figure 7).

Expression of integrin β3 remained unchanged post OVA and Col4 exposure in the trachea. However, it decreased in the OVA-sensitized mice and was increased by 10 mg of Col4 exposure in the lungs (Figure 7).

### Effect of Col4 on EGFR, ERK, and Akt activities in the lungs and trachea

**FIGURE 8:**
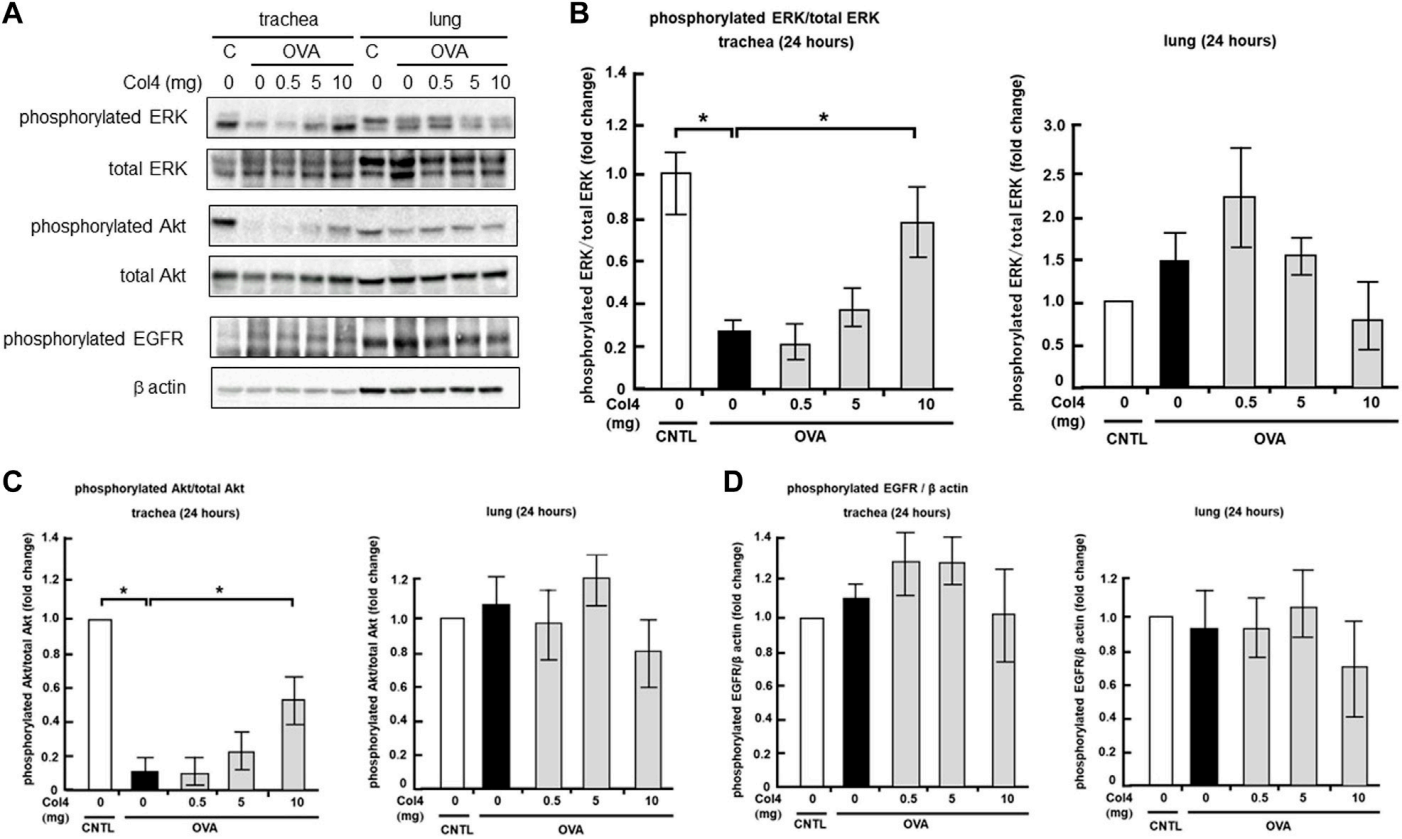
Effects of Col4 on the activities of extracellular signal-regulated kinase (ERK), Akt, phosphorylated epidermal growth factor receptor (EGFR) and β actin levels in ovalbumin (OVA)-sensitized and unsensitized control mice. **(A)** The control mice (C) and OVA-sensitized mice (OVA) were exposed to Col4 at various concentrations (0–10 mg). After 24 h of Col4 exposure, the trachea, and lungs were sampled and phosphorylated ERK, total ERK, phosphorylated Akt, total Akt, phosphorylated EGFR, and β actin levels were detected. Graphs show the relative levels of phosphorylated ERK per total ERK **(B)**, phosphorylated Akt per total Akt **(C)** and phosphorylated EGFR per β actin **(D)** compared with the control. Representative results of three independent experiments are presented. The control mice and the mice treated with 0 mg of Col4 were exposed to PBS alone at the same time.

In the trachea and lung epithelial cell lines, MUC5AC expression was regulated by EGFR, ERK, or Akt activity (Iwashita et al., 2014; Huang et al., 2017; Ito et al., 2019). The effects of Col4 exposure on activities of these regulators were investigated in the trachea and lungs of the OVA-sensitized mice. The level of phosphorylated EGFR did not change significantly as a result of OVA treatment and Col4 exposure (Figure 8). In trachea, the level of phosphorylated ERK decreased in the OVA-sensitized mice but increased following Col4 exposure in a dose-dependent manner. Alterations in the level of phosphorylated ERK were not significant, but a declining tendency was evident in the Col4-exposed OVA-sensitized lungs (Figure 8). In the trachea, the level of phosphorylated Akt also decreased in the OVA-sensitized mice but increased following Col4 exposure in a dose-dependent manner (Figure 8). In the lung tissue, the level of phosphorylated Akt remained unchanged following OVA treatment and Col4 exposure (Figure 8).

## Discussion

Previous studies have revealed that Col4 suppressed MUC5AC expression in the human lung epithelial cell line NCI-H292 and human lung primary cells (Iwashita et al., 2010). In this study, the effect of Col4 on MUC5AC and MUC5B expression was analyzed *in vivo*. The findings revealed that the level of OVA-enhanced MUC5AC protein in BALF and in the lung tissue samples were decreased following Col4 exposure. The reduction in MUC5AC expression by Col4 exposure required 24 h. These results highlighted that adequate dosage and accurate time of exposure were important for Col4 to exert its suppressive effect on MUC5AC expression. The total PAS-stained mucus in the lungs also decreased following Col4 exposure. These results suggest that Col4 reduces MUC5AC expression in the OVA-sensitized lungs *in vivo*.

Conversely, MUC5B expression in the OVA-sensitized mice remained unaffected in both BALF and trachea tissue samples following Col4 exposure. This outcome signifies that the regulation of MUC5B expression differs from that of MUC5AC in the lungs *in vivo*, at least in the case of regulation *via* Col4 exposure.

The expression of integrin α2 and β1 increased in the OVA-sensitized lungs of mice and decreased following Col4 exposure, but was undetected in the trachea. This result suggests that integrin α2 and β1 expression is correlated with MUC5AC secretion and cellular protein levels in the lungs of the OVA-sensitized mice but not in the trachea. We had previously reported that the overexpression of integrin α2 increases the expression of MUC5AC *in vitro* (Iwashita and Murata, 2021). These results allude that the integrin α2β1 complex facilitates higher MUC5AC expression in the OVA-sensitized lungs *in vivo*.

Conversely, OVA treatment reduced integrin β3, but 10 mg of Col4 increased it compared with the OVA treated lungs. Integrin β3 binds to laminin and fibronectin (Barth et al., 2002). Although the relationship between β3, Col4, and MUC5AC secretion is unclear, integrin β3 may be associated with reducing the expression of MUC5AC induced by Col4 exposure *in vivo*. As integrin β3 expression was correlated with MUC5AC secretion in the OVA-sensitized mice, future studies should investigate the relationship between integrin β3 and Col4 and MUC5AC secretion.

Previous studies have shown that the EGFR, Akt, and ERK pathways are involved in MUC5AC expression. This finding implied that the activation of the Akt pathway suppresses MUC5AC expression, whereas activation of the MEK/ERK pathway promotes MUC5AC expression *in vitro* (Iwashita et al., 2014). The levels of phosphorylated EGFR did not change significantly in the tracheas and lungs after OVA treatment and Col4 exposure. The results of this study showed that the levels of phosphorylated Akt were decreased by OVA treatment and increased by Col4 exposure in the trachea. This finding suggests that an increase in the level of phosphorylated Akt suppresses MUC5AC hypersecretion in an EGFR independent manner in the OVA-sensitized trachea.

The levels of phosphorylated ERK were also decreased by OVA treatment and increased by Col4 exposure in the trachea, but showed a declining trend in the Col4-exposed OVA-sensitized lungs. This result signifies that the declining trend in the levels of phosphorylated ERK in the lungs reduces MUC5AC expression in the Col4-exposed OVA-sensitized lungs. In contrast to our results, some reports indicate that Akt and ERK are activated in the lung of OVA-sensitized mice (Alam and Gorska, 2011; Cheng et al., 2011; Ma et al., 2018; Athari, 2019). The ERK is activated in the lung of OVA sensitized C57/BL6 mice (Ma et al., 2018). The Akt is activated in the lung of BALB/c mice at 48 h after the last OVA sensitization (Cheng et al., 2011). However, in both reports, the sampling time and the mouse strain differed from ours. Since ERK and Akt activities were measured at 24 h after exposure to Col4 in our report, further studies must aim to measure their activities at different time points and in different mice strain.

The levels of integrin, phosphorylated ERK, and phosphorylated Akt differed between the lungs and trachea in the OVA-sensitized mice. In the OVA-sensitized lungs, integrin α2 and β1 expression increased but Akt activities remained unchanged. In the OVA-sensitized trachea, integrin β1 was hardly detected but Akt and ERK activities decreased. These different behaviors of integrin subunits, Akt, and ERK between the lungs and trachea and their effects on MUC5AC secretion are complicated. Nevertheless, the changes induced by OVA treatment approached the control level following Col4 exposure. Our results suggest that MUC5AC production is controlled in a tissue-specific manner *in vivo* and that the effect of ERK on MUC5AC secretion is greater in the lungs than in the trachea. In the future, we aim to clarify the relationship between the Col4 effect on MUC5AC expression and integrin intervening pathway, including ERK and Akt regulation pathways *in vivo*.

From the aforementioned results, it could be inferred that Col4 exposure reduces MUC5AC secretion and cellular protein levels in the lungs *in vivo*. This finding implies that Col4 exposure may assist in the treatment of asthma. In human primary cells, repeated additions of low Col4 concentrations reduced MUC5AC expression. Further analyses are required to determine the effect of repeated exposure of small doses of Col4 on MUC5AC expression *in vivo*.

## Conclusion

Exposure of Col4 to OVA-sensitized mice inhibited MUC5AC hypersecretion and expression in the lungs but did not affect MUC5B. The enhanced expression of integrin α2 and β1 proteins in the lungs of OVA-sensitized mice was inhibited after Col4 exposure. In contrast, the suppressed expression of integrin β3 in the lungs of OVA-sensitized mice was increased by Col4 exposure. The levels of Akt and ERK phosphorylation were decreased in the OVA-sensitized mice but increased following Col4 exposure in the trachea. These results suggest that Col4 exposure reduces MUC5AC hypersecretion in the lungs *via* a change in the expression of integrin subunits and in the Akt/ERK activities. Hence, Col4 exposure may contribute to improving asthma treatment.

## Data Availability

The original contributions presented in the study are included in the article/supplementary material, further inquiries can be directed to the corresponding author.
